# Phage Inhibit Pathogen Dissemination by Targeting Bacterial Migrants in a Chronic Infection Model

**DOI:** 10.1128/mBio.00240-17

**Published:** 2017-04-04

**Authors:** Sophie E. Darch, Kasper N. Kragh, Evelyn A. Abbott, Thomas Bjarnsholt, James J. Bull, Marvin Whiteley

**Affiliations:** a Department of Molecular Biosciences, University of Texas at Austin, Austin, Texas, USA; b Institute of Cellular and Molecular Biology, University of Texas at Austin, Austin, Texas, USA; c John Ring LaMontagne Center for Infectious Disease, University of Texas at Austin, Austin, Texas, USA; d Department of Integrative Biology, University of Texas at Austin, Austin, Texas, USA; e Costerton Biofilm Center, Institute of Immunology and Microbiology, University of Copenhagen, Copenhagen, Denmark; f Department of Clinical Microbiology, Copenhagen University Hospital, Copenhagen, Denmark; UT Southwestern Medical Center Dallas

## Abstract

The microbial communities inhabiting chronic infections are often composed of spatially organized micrometer-sized, highly dense aggregates. It has recently been hypothesized that aggregates are responsible for the high tolerance of chronic infections to host immune functions and antimicrobial therapies. Little is currently known regarding the mechanisms controlling aggregate formation and antimicrobial tolerance primarily because of the lack of robust, biologically relevant experimental systems that promote natural aggregate formation. Here, we developed an *in vitro* model based on chronic *Pseudomonas aeruginosa* infection of the cystic fibrosis (CF) lung. This model utilizes a synthetic sputum medium that readily promotes the formation of *P. aeruginosa* aggregates with sizes similar to those observed in human CF lung tissue. Using high-resolution imaging, we exploited this model to elucidate the life history of *P. aeruginosa* and the mechanisms that this bacterium utilizes to tolerate antimicrobials, specifically, bacteriophage. In the early stages of growth in synthetic sputum, planktonic cells form aggregates that increase in size over time by expansion. In later growth, migrant cells disperse from aggregates and colonize new areas, seeding new aggregates. When added simultaneously with phage, *P. aeruginosa* was readily killed and aggregates were unable to form. When added after initial aggregate formation, phage were unable to eliminate all of the aggregates because of exopolysaccharide production; however, seeding of new aggregates by dispersed migrants was inhibited. We propose a model in which aggregates provide a mechanism that allows *P. aeruginosa* to tolerate phage therapy during chronic infection without the need for genetic mutation.

## IMPORTANCE

Bacteria in chronic infections often reside in communities composed of micrometer-sized, highly dense aggregates. A primary challenge for studying aggregates has been the lack of laboratory systems that promote natural aggregate formation in relevant environments. Here, we developed a growth medium that mimics chronic lung infection and promotes natural aggregate formation by the bacterium *Pseudomonas aeruginosa*. High-resolution, single-cell microscopy allowed us to characterize *P. aeruginosa*’s life history—seeding, aggregate formation, and dispersal—in this medium. Our results reveal that this bacterium readily forms aggregates that release migrants to colonize new areas. We also show that aggregates allow *P. aeruginosa* to tolerate therapeutic bacteriophage addition, although this treatment limits *P. aeruginosa* dissemination by targeting migrants.

## INTRODUCTION

Chronic bacterial infections represent a significant health crisis throughout the world, and the growing prevalence of antibiotic resistance in these infections has led to increasing morbidity and mortality (1). With a lack of new antibiotics in the discovery pipeline, alternative therapies are needed to treat the multidrug-resistant pathogens causing chronic infections. One such treatment is the use of the natural viral parasites of bacteria called bacteriophage or phage. Treatment with phage was considered a promising therapeutic option in the early 20th century; however, with the mass production of effective antibiotics after World War II, this line of research was abandoned in all but a few countries (2).

Recently, there has been renewed interest in using phage therapy to treat antimicrobial-resistant pathogens, and its effectiveness has been demonstrated in animal models of septicemia and skin and lung infections (3–5). Phage exhibit the greatest efficacy when added simultaneously with bacterial cells or shortly after the introduction of microbes into an infection site (3, 5–7). Indeed, prophylactic or rapid treatment following infection has been shown to be effective in preventing acute lung infection in mice; however, when treatment was delayed by as little as 2 h following pathogen introduction, efficacy decreased by almost 30% (8). These studies demonstrate that coinoculation of phage with bacterial cells is more effective than the use of phage to treat established infections. The reason for this decreased effect is poorly understood, and a basic question that remains is whether phage therapy should be limited to the early stages of an infection or whether a better understanding of phage-bacterium interactions could be used to more effectively treat established chronic infections.

*In vivo* studies utilizing phage therapy have given hints to phage-bacterium dynamics, but basic details are difficult to uncover in these complex, experimentally restricted systems. For this reason, *in vitro* systems have been used to uncover basic aspects of bacterial growth and resistance. Microfluidic devices, in particular, have been used to study slime-enclosed communities, or biofilms, which are thought to be a major contributor to the persistence of chronic infections. This persistence has been hypothesized to be due to the ability of biofilm bacteria to tolerate host immune functions and antimicrobial therapies (9, 10). However, most *in vitro* biofilm systems lack several important aspects of chronic infections, including the following. The number of cells occupying these *in vitro* biofilms can be 10- to 100-fold greater than those observed in similar volumes *in vivo* (9–11). The nutritional environment used to propagate *in vitro* biofilms does not generally mimic those in chronic infections, although nutrient availability has a profound impact on biofilm development and activity (10, 12). The physical environment, including attachment to plastic or glass and high-flow conditions, does not accurately reflect soft tissue and lung infections (9, 10, 13). For these reasons, there is a need to develop *in vitro* systems that mimic chronic biofilm infections.

To better understand the fundamental aspects of phage-bacterium interactions, we developed a model system that mimics chronic infection by the opportunistic pathogen *Pseudomonas aeruginosa*. *P. aeruginosa* causes a wide range of acute and chronic infections but is most noted as a cause of lung and wound infections, both of which have been proposed as viable phage therapy targets (4, 8, 14). One of the most important lung infections caused by *P. aeruginosa* occurs in individuals with the genetic disorder cystic fibrosis (CF). In the CF lung, *P. aeruginosa* resides in the abundant mucus secretions (here referred to as sputum) produced by individuals with CF. Previously, our lab developed a defined synthetic medium that closely mimics the nutritional composition of human CF sputum (15, 16). Further refinement of this medium by the addition of macromolecules found in CF sputum (mucin, DNA, lipids) revealed that growth of *P. aeruginosa* in this second-generation medium (termed synthetic CF sputum medium 2 [SCFM2]) has genetic requirements similar to those of human sputum (17). Therefore, SCFM2 nutritionally mimics human CF sputum and provides a relevant *in vitro* growth environment for studying chronic *P. aeruginosa* CF infections.

In addition to the nutritional environment, the physical structure of the community is also critical when designing *in vitro* models. For example, *P. aeruginosa* has been shown to grow as micrometer-sized, highly dense aggregates during chronic infection (18), and these aggregates exhibit clinically relevant phenotypes such as antimicrobial tolerance (1, 13, 19). In this study, we show that when grown under static conditions in SCFM2, *P. aeruginosa* grows as aggregates that readily shed bacteria to colonize new areas and seed new aggregates. Viable aggregates of volumes of 34 µm^3^ (~34 cells) and larger are observed following phage treatment and require exopolysaccharide for survival. Though these aggregates display increased tolerance to phage killing, the migrants they release are readily eliminated by phage, thus preventing dissemination and further bacterial spread. These data support a model in which *P. aeruginosa* aggregates are a primary means by which this bacterium tolerates phage therapy during chronic infection and suggest that although phage therapy may not provide a sterilizing effect, it might serve as a viable means to prevent the dissemination of aggregate infections. Moreover, our high-resolution system provides a general model for microbial ecologists to study bacterial life history following disturbances such as antimicrobial therapy.

## RESULTS

### *P. aeruginosa* forms similar-size aggregates in synthetic sputum and CF lung tissue.

**FIG 1:**
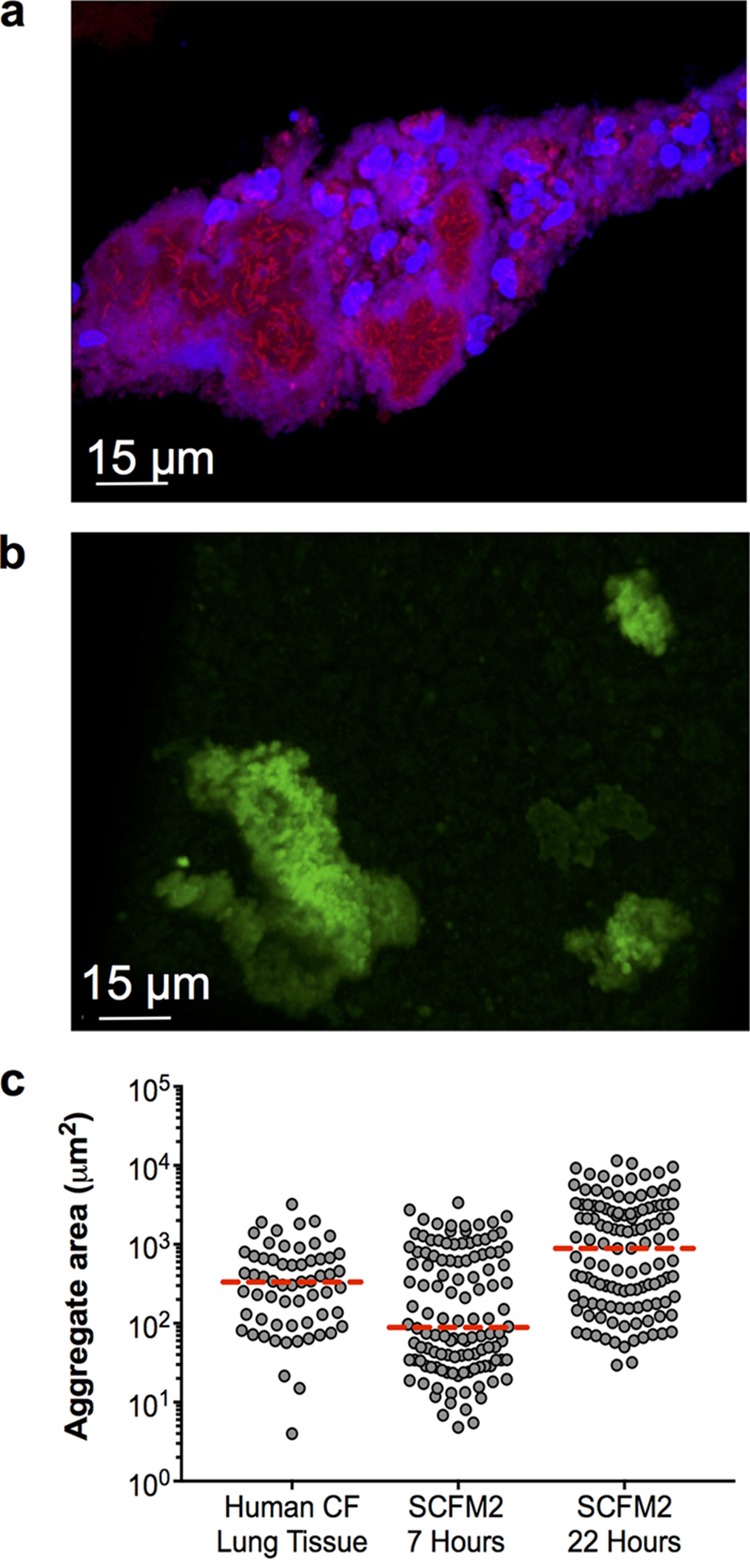
*P. aeruginosa* aggregate sizes are similar in human CF tissue and SCFM2. (a) Micrograph of a *P. aeruginosa* aggregate in an explanted human CF lung tissue section. *P. aeruginosa* was labeled by FISH. *P. aeruginosa* cells are red, and polymorphonuclear leukocytes are blue. (b) Confocal micrograph of a GFP-expressing *P. aeruginosa* aggregate in SCFM2 at 22 h postinoculation. (c) Cross-sectional area of *P. aeruginosa* aggregates from explanted CF lung tissue and *P. aeruginosa* aggregates grown for 7 and 22 h in SCFM2. Each symbol represents an aggregate, and the median is indicated by a broken red line. Measurements were accomplished by manually outlining aggregates, and the area within the outline was calculated with Fiji image analysis software. For these measurements, aggregates were assumed to be entirely filled with cells.

The initial goal of this study was to develop a model that recapitulates important aspects of *in vivo* bacterial growth and physiology and then utilize this model to provide insights into how microbes respond to antimicrobial therapies. The spatial structure of microbial communities has been shown to play a significant role in fitness (20), and recent evidence (18) suggests that *P. aeruginosa* grows as small (micrometer scale), high-density aggregates in human CF infections (Fig. 1a). As these aggregates have been proposed to be critical for *P. aeruginosa* tolerance to host factors and antibiotics (13, 19), we first assessed whether *P. aeruginosa* grows as aggregates in our synthetic sputum (SCFM2). Planktonic (free-swimming) *P. aeruginosa* bacteria expressing the green fluorescent protein (GFP) were added to SCFM2, mixed thoroughly to randomly distribute them, and then added to the wells of chamber slides. At 7 and 22 h postinoculation, confocal laser scanning microscopy (CLSM) was used to generate three-dimensional (3D) images. Our results revealed that *P. aeruginosa* readily forms aggregates in SCFM2 (Fig. 1b). To determine if aggregate sizes were similar in human lung tissue and SCFM2, the area of *P. aeruginosa* aggregates was determined in human CF lung tissue, 7-h SCFM2 aggregates, and 22-h SCFM2 aggregates. Area (rather than volume) measurements were used because *P. aeruginosa* aggregates in human CF lung tissue samples can only be examined by using thin tissue slices. A range of sizes that showed significant overlap for SCFM2 and human CF lung tissues were observed for aggregates under all conditions (Fig. 1c). As observed in the CF lung, *P. aeruginosa* also displayed increased resistance to the antibiotics tobramycin and polymyxin B in SCFM2 (see Fig. S1 in the supplemental material). These results reveal that, in addition to having similar gene expression and gene fitness profiles in SCFM2 and human CF lung tissue (16, 17, 21), *P. aeruginosa* resides in aggregated groups of similar size under both conditions and displays similar antibiotic resistance phenotypes. It is worth noting that aggregate formation in SCFM2 is not limited to strain PAO1 but is also observed in strain PA14 and in CF isolate SED4 (see Fig. S2d and e); it is, however, specific to SCFM2 compared to standard laboratory medium (see Fig. S2a to c).

10.1128/mBio.00240-17.3 FIG S1  SCFM2 promotes *P. aeruginosa* antibiotic resistance. Antimicrobials were applied to luminescent *P. aeruginosa* PAO1, and luminescence was assessed with a luminometer after 60 min of exposure. Decreases in luminescence reflect increases in antimicrobial activity. Data are shown as percentages (referred to as bacterial survival) of the luminescence of antimicrobial-treated cultures relative to the luminescence of a control culture without drug. (a) *P. aeruginosa* survival in SCFM2 and MHB in increasing concentrations of tobramycin sulfate. The 50% lethal dose (LD_50_) for each curve was calculated by performing a nonlinear regression. In SCFM2, the LD_50_ was 0.164 mg/ml, and in MHB, the LD_50_ was 0.009 mg/ml. Each data point represents the average ± the standard error of the mean of four replicates. (b) *P. aeruginosa* survival in SCFM2 and MHB with increasing concentrations of polymyxin B. The LD_50_ for each curve was calculated by performing a nonlinear regression. In SCFM2, the LD_50_ was 0.043 mg/ml, and in MHB, the LD_50_ was 0.002 mg/ml. A randomization test found that the differences between SCFM2 and MHB summed across all doses was statistically significant for both antibiotics (*P* < 0.0001 for both). Each data point represents the average ± the standard error of the mean of four replicates. The standard error bars are often obscured by the data points. Download FIG S1, TIF file, 0.2 MB. Copyright © 2017 Darch et al. 2017 Darch et al. This content is distributed under the terms of the Creative Commons Attribution 4.0 International license.

10.1128/mBio.00240-17.4 FIG S2  SCFM2 promotes aggregation of multiple *P. aeruginosa* strains. *P. aeruginosa* aggregate formation was visualized by phase-contrast microscopy after 7 h in SCFM2 (a), TSB (b), and MHB (c). The scale bar is 15 μm. Experiments were performed in triplicate, and representative micrographs are shown. (d) Growth of *P. aeruginosa* strains PA14 and SED4 in SCFM2 determined by quantification of the total biomass. Biomass was quantified as the number of green fluorescent voxels in GFP-expressing PA14 and SED4. Each data point represents the average ± the standard error of the mean of four replicates. (e) Average *P. aeruginosa* aggregate volume in SCFM2. For these measurements, the total volume of green fluorescent voxels within an aggregate was quantified. Each data point represents the average ± the standard error of the mean of four replicates. Download FIG S2, TIF file, 1 MB. Copyright © 2017 Darch et al. 2017 Darch et al. This content is distributed under the terms of the Creative Commons Attribution 4.0 International license.

### *P. aeruginosa* grows through aggregate expansion and dispersal.

**FIG 2:**
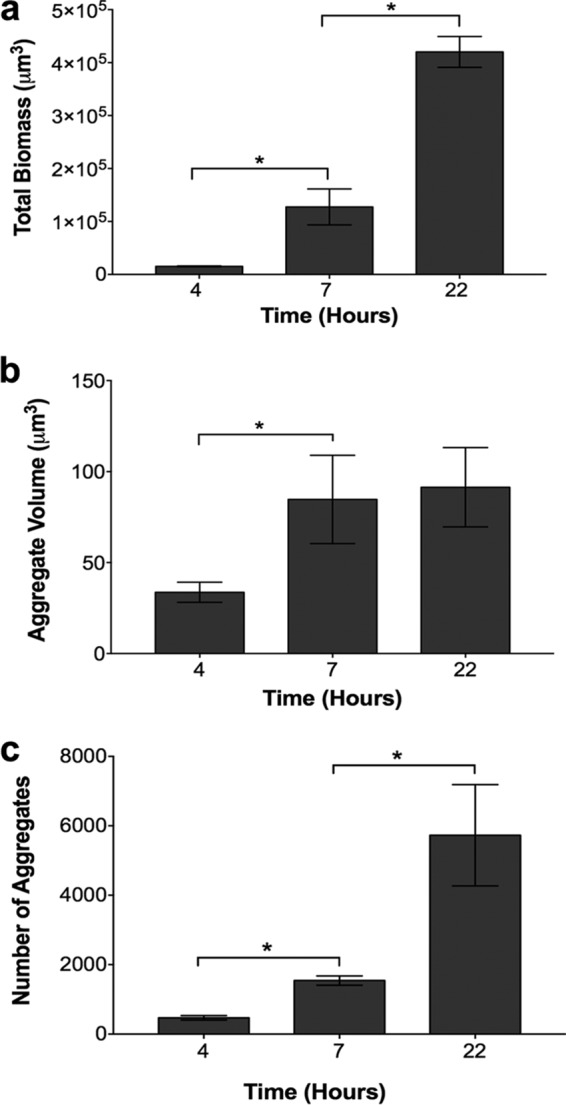
*P. aeruginosa* growth in SCFM2 occurs via aggregate expansion and the formation of new aggregates. (a) Growth of *P. aeruginosa* in SCFM2 determined by quantification of the total biomass. The biomass of *P. aeruginosa* expressing mCherry was quantified as the number of red fluorescent voxels. A heterogeneity test was significant by ANOVA (*F*^2,9^ = 104.5, *P* = 0.0001). An asterisk denotes a statistically significant difference by two-tailed *t* test for comparison of 4- and 7-h data [*t*^(6)^ = 3.298, *P* = 0.0457] and comparison of 7- and 22-h data [*t*^(6)^ = 6.533, *P* = 0.0007]. (b) Average *P. aeruginosa* aggregate volume in SCFM2. To measure the average *P. aeruginosa* aggregate volume in SCFM2, the total volume of red fluorescent voxels within an aggregate was quantified. These measurements differ from those in Fig. 1c, as the high-resolution 3D images used in this analysis allow the precise quantification of cells without the assumption that aggregates are entirely filled with cells. A heterogeneity test was marginally significant by ANOVA (*F*^2,9^ = 4.139, *P* = 0.0531). An asterisk denotes a statistically significant difference by two-tailed *t* test [*t*^(6)^ = 2.568, *P* = 0.04]. (c) Total number of *P. aeruginosa* aggregates in SCFM2. Aggregates were defined as cell clusters of >5.0 µm^3^, and all of the aggregates within each image with dimensions of 135 by 135 by 60 µm were counted. A heterogeneity test was significant by ANOVA (*F*^2,9^ = 10.75, *P* = 0.0041). An asterisk denotes a statistically significant difference by two-tailed *t* test for comparison of 4- and 7-h data [*t*^(6)^ = 7.261, *P* = 0.0014] and comparison of 7- and 22-h data [*t*^(6)^ = 2.854, *P* = 0.0290]. The bars in all of the panels represent the average ± the standard error of the mean of four replicates.

The observation that the aggregate sizes in human CF lung tissue and synthetic CF sputum were similar prompted further investigations of *P. aeruginosa*’s development in this synthetic environment. To accomplish this, we introduced a *P. aeruginosa* strain expressing the red fluorescent protein mCherry into SCFM2 and calculated the total biomass at various time points by quantifying the total red fluorescent voxels in CLSM 3D images. Our results revealed that *P. aeruginosa* grows readily in our static SCFM2 system (Fig. 2a), with an average doubling time of approximately 1.3 h (see Fig. S3a), slightly longer than that observed previously when it was grown with vigorous shaking in synthetic sputum medium (16, 17). To determine if the increase in biomass observed was due to an increased aggregate size (i.e., volume) or an increased number of aggregates, we measured the aggregate volume and quantified the number of aggregates at each time point. The average aggregate volume increased ~3-fold (Fig. 2b; see Fig. S3b) and the number of aggregates increased ~2-fold (Fig. 2c) between 4 and 7 h, while the biomass increased ~8-fold (Fig. 2a).

10.1128/mBio.00240-17.5 FIG S3  *P. aeruginosa* growth in SCFM2 in the absence of shaking. (a) Growth of *P. aeruginosa* in SCFM2 determined by quantification of total biomass. *P. aeruginosa* expressed mCherry, and biomass was quantified as the number of red fluorescent voxels. A generation (doubling) time of 1.3 h was calculated by using data points from 2 to 7 h. Each data point represents the average ± the standard error of the mean of four replicates. (b) *P. aeruginosa* growth in SCFM2 occurs via aggregate expansion and formation of new aggregates. The average *P. aeruginosa* aggregate volume in SCFM2 was determined. For these measurements, the total volume of red fluorescent voxels within an aggregate was quantified. These measurements differ from those in Fig. 1c because the high-resolution 3D images used in this analysis allow precise quantification of cells without the assumption that aggregates are entirely filled with cells. Additional data were obtained at 45 h. An asterisk denotes a statistically significant difference by two-tailed *t* test [*t*^(6)^ = 3.26, *P* = <0.0001]. (c) Maximum *P. aeruginosa* aggregate volumes during growth in SCFM2. Volumes of the 50 largest aggregates are shown for each time point. Each filled circle represents an individual aggregate. The red dashed line is the median of the 50 largest aggregates, and the red filled squares represent mean aggregate volumes, as shown in Fig. 2b. Download FIG S3, TIF file, 0.3 MB. Copyright © 2017 Darch et al. 2017 Darch et al. This content is distributed under the terms of the Creative Commons Attribution 4.0 International license.

Surprisingly, the average aggregate volume remained the same at 7 and 22 h (Fig. 2b) although the biomass increased by ~4-fold. This increase in biomass could be explained by the fact that the number of aggregates also increased by ~4-fold during this time period (Fig. 2c). These data indicate that early *P. aeruginosa* growth (4 to 7 h) is accomplished through increased aggregate volume and increased aggregate numbers, while later stages of growth (7 to 22 h) are accomplished via the formation of new aggregates. After 45 h, the total biomass decreases, as does the average aggregate volume, suggesting that cells either die or become smaller (see Fig. S3). What appears to be occurring is a dynamic process in which established aggregates increase in volume up to a maximum size of ~1,000 µm^3^ (~1,000 rod-shaped cells with a size of 0.8 by 2 µm, see Fig. S3c), while the average size is maintained much smaller at ~85 µm^3^ because new aggregates are being continually formed in ever larger numbers, offsetting the continued growth of the large aggregates. The process may resemble the demographic phenomenon of a stable age distribution.

### Many* P. aeruginosa* aggregates survive phage treatment

On the basis of the observation that *P. aeruginosa* forms similar-size aggregates (Fig. 1) and displays similar physiology in SCFM2 and the CF lung (see Fig. S1) (15–17), we next examined the utility of this model for assessment of the efficacy of phage therapeutics. The goal was to provide insights into why phage therapy shows limited efficacy when used to treat established infections while exhibiting high efficacy when used prophylactically (4,7,8). For this study, we used a cocktail of two lytic phage that have been proposed to utilize different receptors and were previously used in phage therapy studies (14). Phage were added at a 2:1 ratio with bacteria, and this level of phage was sufficient to sterilize a *P. aeruginosa* planktonic culture grown in laboratory medium within 18 h (~7-log reduction, with cell elimination to the limit of detection; see Fig. S5a). As most phage therapy studies have shown maximum effectiveness both *in vitro* and *in vivo* when phage are coinoculated with bacteria, we initiated our studies by adding the phage cocktail simultaneously with *P. aeruginosa* into SCFM2 and examining the biomass for 18 h. Coinoculation with phage resulted in an ~100-fold decrease in *P. aeruginosa* biomass after 18 h (see Fig. S5b), further supporting the effectiveness of phage as prophylactic therapeutics.

**FIG 3:**
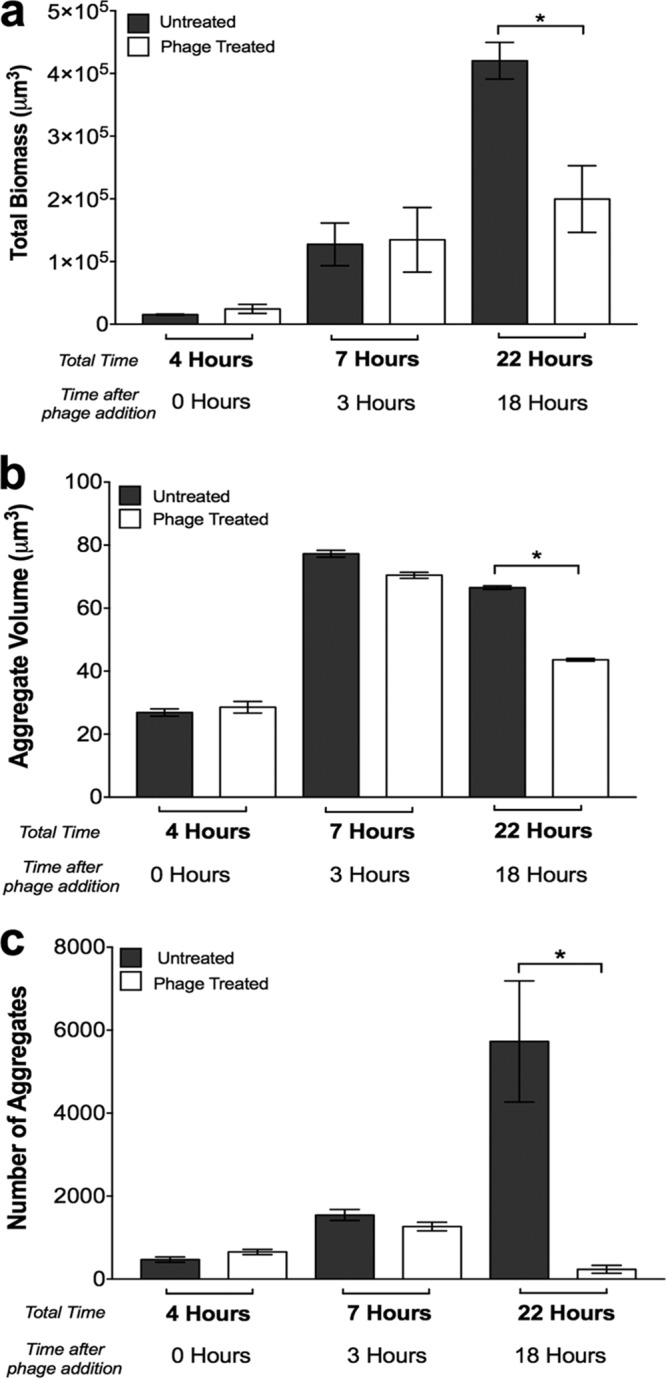
Phage have a minimal impact on preformed *P. aeruginosa* aggregates but prevent the formation of new aggregates. (a) Average *P. aeruginosa* growth (total biomass) in SCFM2 in the presence and absence of phage. *P. aeruginosa* was allowed to form aggregates for 4 h before phage addition, and the biomass was calculated 3 and 18 h after phage addition. A heterogeneity test was significant, as determined by ANOVA (*F*^2,9^ = 1.3, *P* = 0.0503). An asterisk denotes a statistically significant difference by two-tailed *t* test [*t*^(6)^ = 3.633, *P* = 0.0170]. (b) *P. aeruginosa* average aggregate volumes in SCFM2 in the presence and absence of phage. *P. aeruginosa* was allowed to form aggregates for 4 h before phage addition, and the aggregate volume was calculated 3 and 18 h after phage addition. A heterogeneity test was significant, as determined by ANOVA (*F*^2,9^ = 4.143, *P* = 0.053). An asterisk denotes a statistically significant difference by two-tailed *t* test [*t*^(6)^ = 29.21, *P* < 0.0001]. (c) Total numbers of *P. aeruginosa* aggregates in SCFM2 in the presence and absence of phage. Aggregates were defined as cell clusters of >5.0 µm^3^, and all of the aggregates within each image with dimensions of 135 by 135 by 60 µm were counted. A heterogeneity test was significant by ANOVA (*F*^2,9^ = 34.208, *P* = <0.0004). An asterisk denotes a statistically significant difference between groups at 22 h by two-tailed *t* test [*t*^(6)^ = 3.75, *P* = 0.0095]. The bars in all of the panels represent the average ± the standard error of the mean of four replicates

To examine the impact of phage addition on microbial communities growing as aggregates, we allowed *P. aeruginosa* to form aggregates in synthetic medium for 4 h (4 h, Fig. 3a) and then treated these communities with the phage cocktail. Phage had no significant impact on *P. aeruginosa* biomass at 3 h after phage addition (7 h, Fig. 3a), while there was a significant decrease in the biomass of phage-treated communities 18 h after phage addition (22 h, Fig. 3a). However, unlike the coinoculation experiments (see Fig. S5B), communities allowed to develop for 4 h, and therefore containing *P. aeruginosa* aggregates, retained high levels of viable biomass (~50% of untreated communities, Fig. 3a; see Fig. S5c), suggesting that these communities are more recalcitrant to phage treatment. Examination of aggregate volumes revealed that, similar to biomass (Fig. 3a), phage treatment had no significant impact on the average volume of individual aggregates 3 h after phage addition (7 h, Fig. 3b). Indeed, average aggregate volumes increased ~3-fold between 4 and 7 h under both phage-treated and untreated conditions (Fig. 3b), indicating that the ~4-fold increase in biomass observed during this time was, in part, due to an increased volume of individual aggregates. Aggregate volume and number did show a significant decline at the latest time point in phage-treated cultures (22 h, Fig. 3b and c), indicating that while aggregates are clearly more tolerant to phage, they do potentially display some susceptibility after longer exposure times. These results reveal that preincubation of *P. aeruginosa* for 4 h in SCFM2 significantly enhances tolerance to phage treatment, and a significant portion of the increased biomass observed following phage treatment is due to increased aggregate volume. Of note, treatment of aggregates with individual phage did not cause a significant reduction in biomass after 22 h, indicating that the addition of both phage is required (see Fig. S4).

10.1128/mBio.00240-17.6 FIG S4  Individual phage did not significantly reduce the total biomass of preformed *P. aeruginosa* aggregates. The average *P. aeruginosa* growth (total biomass) in SCFM2 in the presence and absence of phage was determined. Phage were added individually or in combination. *P. aeruginosa* was allowed to form aggregates for 4 h before phage addition, and the biomass was calculated 3 and 18 h after phage addition. Differences between untreated cultures at 22 h and individual phage 8-16 and 8-17 were not significant, as determined by two-tailed *t* test [*t*^(6)^ = 2.091 (*P* = 0.147) and *t*^(6)^ = 1.679 (*P* = 0.322), respectively]. An asterisk denotes a statistically significant difference by two-tailed *t* test [*t*^(6)^ = 3.633, *P* = 0.0170]. Download FIG S4, TIF file, 0.2 MB. Copyright © 2017 Darch et al. 2017 Darch et al. This content is distributed under the terms of the Creative Commons Attribution 4.0 International license.

10.1128/mBio.00240-17.7 FIG S5  Addition of phage sterilizes planktonic *P. aeruginosa* grown in laboratory medium while delayed treatment leaves viable biomass in the form of aggregates. *P. aeruginosa* was grown in TSB with shaking. Phage were added simultaneously with bacterial cells (two phage per bacterium) and incubated for 18 h. At 18 h, samples were taken, serially diluted, and plated onto TSB agar plates to determine the number of CFU per milliliter. Comparison was made with an untreated control. Bars represent the average ± the standard error of the mean of three replicates. The vertical dashed line is the limit of detection, and ND means no data above the limit of detection. (b) Prophylactic phage treatment reduces the *P. aeruginosa* biomass in SCFM2. Phage and *P. aeruginosa* were added simultaneously (two phage per bacterium) to SCFM2, followed by biomass quantification at 2, 6, and 18 h. *P. aeruginosa* expressed mCherry, and biomass was quantified as the number of red fluorescent voxels. Bars represent the average ± the standard error of the mean of four replicates. An asterisk denotes a statistically significant difference between untreated and treated groups, as determined by a two-tailed *t* test at 6 h [*t*^(6)^ = 3.47, *P* = 0.0133] and 18 h [*t*^(6)^ = 7.25, *P* = 0.0003]. (c) *P. aeruginosa* aggregates treated with phage are viable. Viability of *P. aeruginosa* aggregates in SCFM2 ± phage treatment after 22 h. Aggregates were allowed to develop for 4 h before the addition of phage. Eighteen hours after phage addition, samples were taken, serially diluted, and plated onto TSB agar to determine the number of CFU per milliliter. Bars represent the average ± the standard error of the mean of three replicates. An asterisk denotes a statistically significant difference by a two-tailed *t* test [*t*^(3)^ = 3.75, *P* = 0.004]. Download FIG S5, TIF file, 0.2 MB. Copyright © 2017 Darch et al. 2017 Darch et al. This content is distributed under the terms of the Creative Commons Attribution 4.0 International license.

### *P. aeruginosa* exopolysaccharides provide protection against phage.

**FIG 4:**
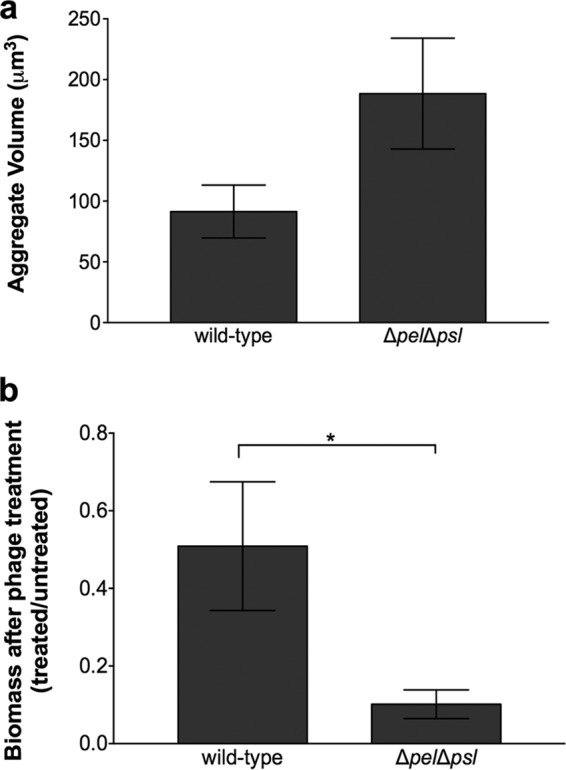
*P. aeruginosa* Pel and Psl exopolysaccharides are not required for aggregate formation but provide protection from phage killing. (a) Average aggregate volumes of wild-type *P. aeruginosa* and *P. aeruginosa* Δ*pel*Δ*psl* in SCFM2 in the presence and absence of phage. *P. aeruginosa* was allowed to form aggregates for 4 h in SCFM2 before phage addition, and the aggregate volume was assessed 18 h later. Bars represent the average ± the standard error of the mean of four replicates. Aggregate volumes were not statistically significantly different by ANOVA (*F*^1,6^ = 2.28, *P* = 0.1819). (b) Wild-type *P. aeruginosa* and *P. aeruginosa* Δ*pel*Δ*psl* biomasses following phage treatment. Wild-type *P. aeruginosa* and *P. aeruginosa* Δ*pel*Δ*psl* were allowed to form aggregates for 4 h in SCFM2 before phage addition, and their biomasses were assessed 18 h later. Bars represent the average fraction of biomass present in phage-treated and untreated samples. Error bars show the standard error of the mean of four replicates. An asterisk denotes a statistically significant difference between wild-type *P. aeruginosa* and *P. aeruginosa* Δ*pel*Δ*psl*, as determined by a two-tailed *t* test [*t*^(12)^ = 2.4656, *P* = 0.0149].

*In vitro* biofilm studies have shown that bacterial exopolysaccharides can, in some but not all cases, provide protection against phage (22–24); thus, we next examined whether *P. aeruginosa* exopolysaccharides protected aggregates from phage-mediated killing. *P. aeruginosa* PAO1 has the capacity to produce at least three extracellular polysaccharides; alginate, Pel, and Psl. Since alginate is not produced by strain PAO1 in biofilms (25), we focused on Pel and Psl in these experiments. We compared the aggregate formation and phage tolerance of wild-type *P. aeruginosa* to those of an isogenic *P. aeruginosa* mutant that cannot produce Pel and Psl. Elimination of Pel and Psl did not impact the ability of *P. aeruginosa* to produce aggregates (Fig. 4a), and the average size of these aggregates was, on average, larger than that of those formed by the wild type (although the difference was not statistically significant, *P* = 0.18). However, aggregates formed by the Pel/Psl mutant were significantly more susceptible to killing by phage than those formed by exopolysaccharide-producing wild-type *P. aeruginosa*, as determined by calculation of the total biomasses of untreated and phage-treated cultures (Fig. 4b). Similar to the Pel/Psl mutant, *P. aeruginosa* mutants unable to produce individual polysaccharides formed aggregates; however, these mutants did not display increased susceptibility to phage killing (see Fig. S6). These data indicate that PAO1 exopolysaccharides are not required for aggregate formation in SCFM2 and that Pel and Psl collectively protect aggregates against phage.

10.1128/mBio.00240-17.8 FIG S6  *P. aeruginosa* exopolysaccharides are not required for aggregate formation but provide protection from phage killing. (a) Average aggregate volumes of wild-type *P. aeruginosa* and *P. aeruginosa* Δ*pel*Δ*psl*, Δ*pel*, Δ*psl*, and Δ*algD* in SCFM2 in the presence and absence of phage. *P. aeruginosa* was allowed to form aggregates for 4 h in SCFM2 before phage addition, and the aggregate volume was assessed 18 h later. Bars represent the average ± the standard error of the mean of four replicates. (b) Wild-type *P. aeruginosa* and *P. aeruginosa* Δ*pel*Δ*psl*, Δ*pel*, Δ*psl*, and Δ*algD* biomasses following phage treatment. Wild-type *P. aeruginosa* and all of the isogenic deletion mutants tested were allowed to form aggregates for 4 h in SCFM2 before phage addition, and their biomasses were assessed 18 h later. Bars represent the average fractions of biomass present in phage-treated and untreated samples. An asterisk denotes a statistically significant difference between wild-type *P. aeruginosa* and *P. aeruginosa* Δ*pel*Δ*psl*, as determined by a two-tailed *t* test [*t*^(12)^ = 2.4656, *P* = 0.0149]. Comparisons of individual Δ*pel*, Δ*psl*, and Δ*algD* mutants with the wild type showed no significant differences, as determined by a two-tailed *t* test (*P* = 0.23, *P* = 0.52, *P* = 0.25, respectively). Error bars show the standard error of the mean of four replicates. Download FIG S6, TIF file, 0.4 MB. Copyright © 2017 Darch et al. 2017 Darch et al. This content is distributed under the terms of the Creative Commons Attribution 4.0 International license.

### Phage target bacterial migrants.

**FIG 5:**
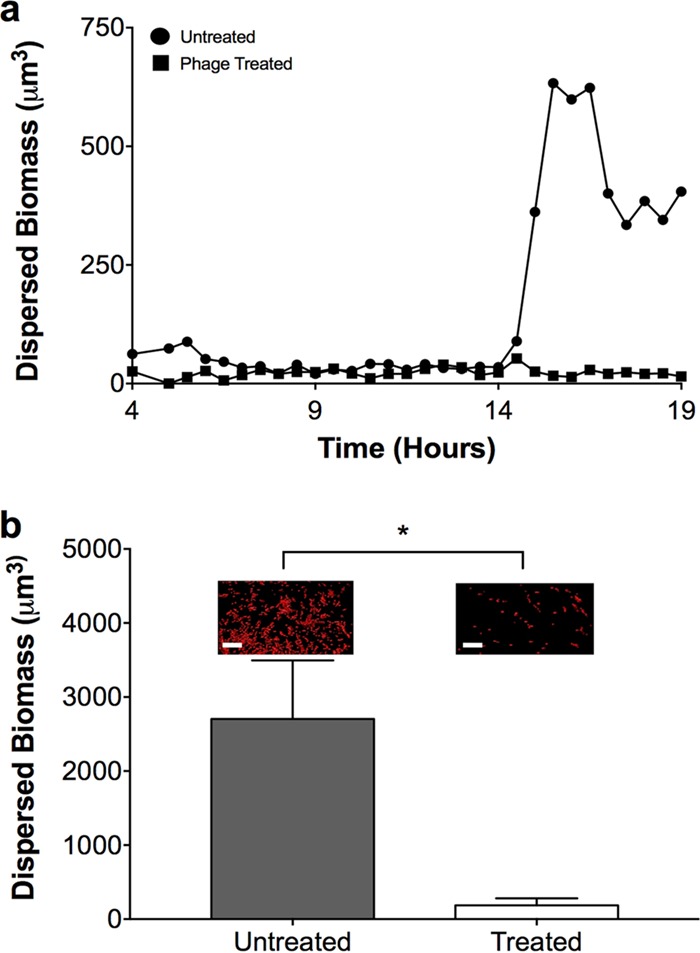
Phage reduce bacterial migrant numbers and prevent *P. aeruginosa* dispersal. (a) Dispersed *P. aeruginosa* biomasses during growth in SCFM2 in the presence (■) and absence (●) of phage. *P. aeruginosa* was allowed to form aggregates for 4 h in SCFM2 before phage addition, and the dispersed biomass was assessed every 30 min for 15 h. Dispersed biomass was defined as individual cell clusters of ≤5.0 µm^3^, which corresponds to about one to five bacteria. Data from a single replicate are presented for clarity. Three additional replicates are shown in Fig. S7. (b) Dispersal of *P. aeruginosa* in SCFM2. *P. aeruginosa* was allowed to form aggregates for 4 h in SCFM2 and then overlaid with sterile SCFM2 (untreated) or SCFM2 containing phage (treated). After 6 h, the amount of dispersed biomass in the upper SCFM2 layer was determined as the number of red fluorescent voxels and is represented as the mean biomass ± the standard error of the mean of four replicates. Representative micrographs of dispersed biomass are shown above each bar. Scale bar is 20 µm. An asterisk denotes a statistically significant difference between untreated and treated samples, as determined by a two-tailed *t* test [*t*^(6)^ = 2.5, *P* = 0.044].

Why does the biomass of *P. aeruginosa* aggregate communities increase during the first 3 h following phage treatment but not the next 18 h (Fig. 3a)? Since early bacterial growth (4 to 7 h, Fig. 2) is accomplished through increases in aggregate volume and aggregate numbers, while later growth (7 to 22 h, Fig. 2) is accomplished via the formation of new aggregates, we hypothesized that phage inhibit the formation of new aggregates by targeting migrant bacteria that disperse from existing aggregates. To test this hypothesis, we used CLSM to assess dispersed and aggregated biomasses in the presence and absence of phage. We predicted that if phage target bacterial migrants, fewer dispersed cells would be observed in phage-treated samples than in untreated samples. For these experiments, dispersed biomass was considered to be volumes of ≤5 µm^3^, while aggregated groups were volumes of >5 µm^3^. These values were chosen on the basis of the idea that *P. aeruginosa* could be motile and disperse as individual cells or small groups of 5 µm^3^ (~5 cells) but not larger groups. As in previous experiments, *P. aeruginosa* was first allowed to form aggregates for 4 h in SCFM2 before phage addition, and CLSM was performed every 30 min for 15 h to provide a refined assessment of aggregate formation. In the absence of phage, dispersed cells were observed throughout the experiment (Fig. 5a), although the amount of dispersed biomass differed between replicates (see Fig. S7). There were also several time points when dispersed biomass increased sharply within a 1-h period, often >100-fold (Fig. 5a; see Fig. S7 and Video S1). In contrast, the presence of phage significantly decreased the levels of dispersed cells and no sharp increases in dispersal were observed; however, small numbers of dispersed cells were always observed in these cultures (Fig. 5a; see Fig. S7 and Video S2). Collectively, these results suggest that phage impact *P. aeruginosa* colonization of new areas by decreasing the number of dispersed cells.

10.1128/mBio.00240-17.1 VIDEO S1  The *P. aeruginosa* life cycle in SCFM2 involves aggregate formation and dispersal. *P. aeruginosa* was allowed to form aggregates for 4 h in SCFM2 and then imaged via CLSM every 30 min for 15 h. Isosurfaces were produced to identify aggregated and dispersed biomasses with Imaris image analysis software. Aggregate biomass is shown in red and corresponds to cell clusters of >5.0 μm^3^. Dispersed cells are shown in green and are defined as cell volumes of ≤5.0 μm^3^ (corresponding to about one to five bacteria). Data from Fig. S6c and Fig. 5a were used to generate this video. Download VIDEO S1, AVI file, 7.9 MB. Copyright © 2017 Darch et al. 2017 Darch et al. This content is distributed under the terms of the Creative Commons Attribution 4.0 International license.

10.1128/mBio.00240-17.2 VIDEO S2  Phage target *P. aeruginosa* migrants. *P. aeruginosa* was allowed to form aggregates for 4 h in SCFM2, at which time phage were added and then imaged via CLSM every 30 min for 15 h. Isosurfaces were produced to identify aggregated and dispersed biomass with Imaris image analysis software. Aggregate biomass is shown in red and corresponds to cell clusters of >5.0 μm^3^. Dispersed cells are shown in green and are defined as cell volumes of ≤5.0 μm^3^ (corresponding to about one to five bacteria). Data from Fig. S6c and Fig. 5a were used to generate this video. Download VIDEO S2, AVI file, 4.4 MB. Copyright © 2017 Darch et al. 2017 Darch et al. This content is distributed under the terms of the Creative Commons Attribution 4.0 International license.

10.1128/mBio.00240-17.9 FIG S7  Phage reduce bacterial *P. aeruginosa* migrants in SCFM2 (a to d). Four replicates showing dispersed *P. aeruginosa* biomass during growth in SCFM2 in the presence (■) and absence (●) of phage are shown. *P. aeruginosa* was allowed to form aggregates for 4 h in SCFM2 before phage addition, and dispersed biomass was assessed every 30 min for 15 h. Dispersed biomass was defined as individual cell clusters of ≤5.0 µm^3^. Data from panel c are shown in Fig. 5A. Download FIG S7, TIF file, 0.4 MB. Copyright © 2017 Darch et al. 2017 Darch et al. This content is distributed under the terms of the Creative Commons Attribution 4.0 International license.

### Phage reduce aggregate dispersal.

To further test the hypothesis that phage target bacterial migrants, we developed an aggregate dispersal assay to directly assess the impact of phage treatment on *P. aeruginosa* dispersal. This assay begins by forming *P. aeruginosa* aggregates for 4 h, followed by the addition of sterile SCFM2 on top of the SCFM2 containing aggregates. This assay measures dispersal by examining the appearance of *P. aeruginosa* biomass in the overlying uninoculated SCFM2 after 6 h. In the absence of phage, *P. aeruginosa* was readily observed in the uninoculated SCFM2, as both single cells (6% of the dispersed biomass, defined as sizes of 0.5 to 3 µm^3^) and as larger aggregates; however, addition of phage significantly reduced dispersal (Fig. 5b). As expected, *P. aeruginosa* was observed in the bottom SCFM2 layer following phage treatment (see Fig. S8a) and most of this biomass was in the form of aggregates (Fig. S8b), indicating that the lack of dispersal was not due to phage-mediated aggregate killing. These data reveal that *P. aeruginosa* migrates and colonizes new sites in SCFM2 and does this, at least in part, as single cells. In addition, these results indicate that while phage do not eliminate all of the established aggregates, they do prevent *P. aeruginosa* dispersal.

10.1128/mBio.00240-17.10 FIG S8  *P. aeruginosa* aggregates survive phage treatment in the dispersal assay. (a) Total biomass remaining in the bottom layer of the dispersal assay following phage exposure. *P. aeruginosa* was allowed to form aggregates for 4 h in SCFM2 and then overlaid with sterile SCFM2 (untreated) or SCFM2 containing phage (treated). After 6 h, the amount of biomass in the lower SCFM2 layer was determined as the number of red fluorescent voxels and is represented as the mean biomass ± the standard error of four replicates. An asterisk denotes a statistically significant difference by a two-tailed *t* test [*t*^(6)^ = 2.357, *P* = 0.0286]. (b) The biomass remaining in the bottom layer of the dispersal assay after 6 h was determined as migrant (≤5.0 µm^3^) or aggregate (>5.0 µm^3^), and the migrant-to-aggregate ratios in the presence and absence of phage were calculated. The biomass was determined as the number of red fluorescent voxels and is represented as the mean biomass ± the standard error of four replicates. An asterisk denotes a statistically significant difference between the untreated and phage-treated groups, as determined by a one-tailed nonparametric median test (*P* = 0.0143). Download FIG S8, TIF file, 0.2 MB. Copyright © 2017 Darch et al. 2017 Darch et al. This content is distributed under the terms of the Creative Commons Attribution 4.0 International license.

## DISCUSSION

The inability to combat chronic bacterial infections with modern therapeutics remains a significant problem throughout the world. While it was long ago proposed that bacteria exist as biofilms in chronic infections, it is now clear that in chronic wounds and CF sputum, they grow as micrometer scale, highly dense aggregates (10, 13, 18, 26). While the biology of bacterial aggregates is poorly understood, they do display characteristic phenotypes, including enhanced antibiotic tolerance and resilience to host immune factors, that have been observed in conventional *in vitro* biofilms (19, 27–29). One of the primary challenges to studying aggregates is the lack of robust, biologically relevant experimental systems for creating them. Here, we show that *P. aeruginosa* forms aggregates readily in a synthetic CF sputum medium that chemically mimics human CF sputum (Fig. 1). This medium contains high levels of mucin, lipid, and DNA, and these components appear to be key to aggregate formation, as synthetic sputum without these components does not promote aggregation (15, 16). However, it should be noted that *P. aeruginosa* is not restricted to aggregate growth in SCFM2, as single cells and small groups of cells are observed throughout growth (Fig. 5a; see Fig. S7). Indeed, while there was variability in the timing and magnitude, there are clear instances in which the number of single cells and small groups of cells increased dramatically (Fig. 5a; see Fig. S7). These data support a model of aggregate formation in which planktonic cells form aggregates that release migrants that travel to and form aggregates in uncolonized areas. Of course, while we cannot eliminate the possibility that aggregates are formed by other mechanisms such as splitting of existing aggregates, our data provide strong evidence that seeding of new aggregates by migrants is a critical mechanism for bacterial spread in our model. It is also important to point out that the use of a non-CF-adapted *P. aeruginosa* strain for these studies was intended to mimic the initial stages of sputum colonization. Future studies will examine the life history of CF-adapted strains or more diverse communities.

Interestingly, aggregate formation in SCFM2 does not require the *P. aeruginosa* exopolysaccharides Pel and Psl (Fig. 4a). Pel and Psl have both been shown to be critical for biofilm formation *in vitro*, aiding surface attachment and maintaining biofilm structural integrity (30); however, those studies have generally examined biofilm formation on glass surfaces in flowing systems with laboratory medium as the nutritional source. Thus, it may not be surprising that Pel and Psl are not important for aggregate formation in viscous, static SCFM2. We speculate that polymers such as mucin and DNA in the synthetic medium may provide structural scaffolds for aggregate formation, thus eliminating the need for exopolysaccharide production. However, these components were not sufficient for protection against phage, as Pel and Psl were critical determinants of aggregate tolerance (Fig. 4b). The reduction in exopolysaccharide mutant biomass after treatment with phage indicates that the mechanism of aggregate tolerance is, at least in part, due to exopolysaccharide production. Exopolysaccharides have been shown to be important for phage tolerance in some, but not all, bacteria (22–24), although these studies have not focused on aggregates. Another possibility is that the presence of Pel and Psl impacts the accessibility or level of the phage receptor. Collectively, these results suggest that phage containing exopolysaccharide-degrading enzymes or other strategies targeting exopolysaccharide biosynthesis or stability may be a viable means of enhancing phage effectiveness against bacterial aggregates. Such strategies using glycoside hydrolases to target *P. aeruginosa* exopolysaccharides have shown promise in enhancing the susceptibility of biofilms to neutrophil killing (31).

A large number of phage therapy studies show high efficacy when phage are administered prophylactically with low-density planktonic bacteria; however, treatment of established infections often results in a minimal impact on bacterial numbers and disease outcomes (4, 8, 32). High-density biofilm growth has been hypothesized as a mechanism of antimicrobial protection in established infection, and recent evidence supporting this hypothesis shows that social interactions during high-density growth are key to bacterial phage tolerance (33). While it is clear that bacteria do not grow as traditional “mushroom” biofilms in many infections, including CF (13, 18), our discovery that aggregates with volumes of >34 µm^3^ are present following phage treatment and even grow in the presence of phage (Fig. 3) provides strong experimental evidence that aggregates must reach a critical mass to tolerate phage. In addition to protection by exopolysaccharides, the concept of a “critical” aggregate size provides new insights into the mechanisms microbes use to evade phage killing. It could be argued that the resistance of aggregates to phage may be due to the increase in biomass that occurs during aggregate formation in our experimental system. However, we do not favor this idea since the exopolysaccharide mutant, which increases similarly in biomass, is highly susceptible to phage (Fig. 4b). Thus, the levels of phage utilized in our experimental design are sufficient to reduce aggregate population numbers in the time frame of our experiment. Collectively, our results suggest that phage therapy will likely have limited utility in eliminating bacterial infections caused by aggregates. Nevertheless, the observation that treatment with phage reduces the number of bacterial migrants and prevents the colonization of new areas (Fig. 5) suggests a potential role for these treatments in preventing the spread of aggregate infections.

**FIG 6:**
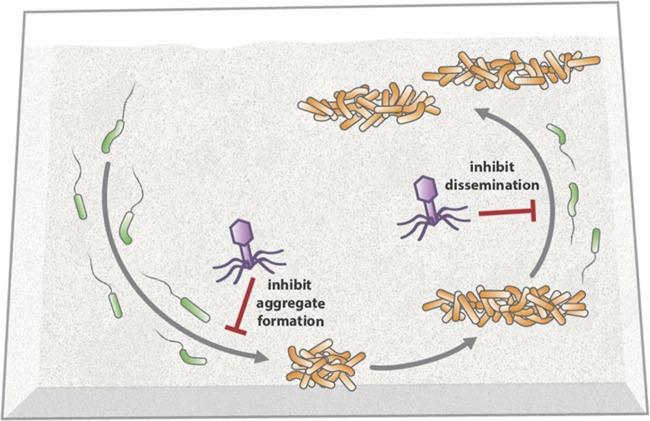
Model of *P. aeruginosa* life history and phage susceptibility in SCFM2. Upon entering synthetic sputum, planktonic *P. aeruginosa* (shown in green on the left side) readily forms aggregates (shown in orange) within 4 h that increase in volume by 7 h. At 7 h, planktonic migrants are released from aggregates (shown in green on the right side) that move to unoccupied areas and ultimately seed new aggregates. Phage block planktonic *P. aeruginosa* from forming aggregates (inhibit aggregate formation) and dispersed cells from forming new aggregates (inhibit dissemination).

On the basis of the results of this study, we propose a model of the life history of *P. aeruginosa* in synthetic sputum (Fig. 6) that initiates with planktonic cells establishing aggregates within 4 h that increase in size at 7 h. Subsequent growth of the community is primarily mediated by the release of migrants from aggregates that travel to uncolonized regions and develop into aggregates. The effect of phage treatment on the bacterial community is dependent on the developmental stage, as phage can largely prevent initial aggregate formation by planktonic cells and the formation of new aggregates by migrants while having less of an effect on established aggregates (Fig. 6). In retrospect, targeting of *P. aeruginosa* migrants is not a surprise, as *P. aeruginosa* migration is accomplished, at least in part, by single planktonic cells that should be susceptible to phage attack (Fig. 5a). An alternative possibility is that phage addition simply causes *P. aeruginosa* aggregates not to release migrants. We find this unlikely, given the fact that single cell migrants were observed in both our phage-treated aggregate dispersal experiment (Fig. 5b) and our time-lapse experiment (Fig. 5a; see Video S2). Ultimately, our results provide new insights into the life history of *P. aeruginosa* during the colonization of synthetic sputum, as well as a robust model for studying the mechanisms used by *P. aeruginosa* to tolerate environmental insults such as phage therapy.

## MATERIALS AND METHODS

### Strains and media used.

Experiments were performed with wild-type *P. aeruginosa* strain PAO1 carrying either GFP-expressing plasmid pMRP9-1 (34) (Fig. 1b) or mCherry-expressing plasmid pGJB5 (all other figures) (28). Antibiotic resistance studies were performed with PAO1 carrying the luminescence reporter plasmid pQF50-lux (35). To determine if aggregate formation was strain specific, *P. aeruginosa* strain PA14 and LESB58 clinical isolate SED4 (36) carrying pMRP9-1 (34) were used. Studies examining the importance of exopolysaccharides for aggregate formation and phage tolerance were performed with* P. aeruginosa* PAO1 Δ*pel* Δ*psl* and PAO1 Δ*psl* (25) containing pGJB5 (28) and PAO1 Δ*pel* and PAO1 Δ*algD* (25) containing pMRP9-1 (34). Overnight cultures were grown in tryptic soy broth (TSB) at 37°C with shaking at 200 rpm. All washing steps were performed with 1× phosphate-buffered saline (PBS), pH 7.0. Aggregate studies were performed with SCFM2 (17).

### Aggregate formation in SCFM2.

*P. aeruginosa* carrying pMRP9-1 or pGJB5 was grown overnight in TSB. Cells were washed twice and resuspended in PBS. The optical density at 600 nm (OD_600_) was measured with a spectrophotometer, and washed bacterial cultures were inoculated into SCFM2 at an OD_600_ of 0.05 (~10^7^ CFU/ml). Cultures were vortexed for 5 to 10 s to disperse bacterial cells in SCFM2. Five hundred microliters of inoculated SCFM2 was then transferred into each well of four-well microchamber slides (Lab-Tek; Nunc) and incubated under static conditions at 37°C.

### Aggregate formation in TSB and MHB.

Experiments were prepared as described in the previous paragraph, but cells were inoculated into either Mueller-Hinton broth (MHB) or TSB and incubated under static conditions at 37°C.

### MIC assay.

The antibiotics tobramycin sulfate and polymyxin B were prepared in stock solutions of 10 mg/ml and diluted to the required concentrations in sterile distilled water. Two hundred microliters of SCFM2 or MHB containing antibiotic was added to the appropriate wells of a 96-well microtiter plate and serially diluted 2-fold across adjacent wells of the plate into 100 μl of appropriate medium, producing a concentration range of 2.5 to 0.005 mg/ml. One hundred microliters of washed *P. aeruginosa* PAO1 carrying plasmid pQF50-lux was adjusted to a standardized OD_600_ of 0.05 in either medium. One hundred microliters of each isolate was then added to each appropriate well. Suitable negative controls containing no antimicrobial and isolate-free wells were included on every microtiter plate. Cultures were incubated statically for 60 min, after which luminescence was measured with a Luminoskan Ascent microplate luminometer as a proxy for cell viability.

### Phage treatment.

Two bacteriophage belonging to *Pseudomonas* bacteriophage families NP3 (ATCC 12175-B1) and PA2 (ATCC 14203-B1) were used in this study (14). Phage treatment was applied as a cocktail containing equal amounts of each phage to SCFM2 containing *P. aeruginosa* carrying pGJB5 to recapitulate prophylactic therapy or treatment of an established infection. For prophylactic treatment, immediately following the addition of SCFM2 inoculated with *P. aeruginosa* to the microchamber well, the phage cocktail was added at a ratio of 2:1 (phage to bacterial cells) to the center top of SCFM2 and agitated slightly. Cultures were then imaged by CLSM at 2, 6, and 18 h posttreatment. For the treatment of an established infection,* P. aeruginosa* aggregates were allowed to develop for 4 h in a microchamber well containing SCFM2, followed by phage addition as described above for prophylactic treatment. The volume of phage needed to maintain a 2:1 ratio of phage to bacterial cells was calculated by using the known total biomass of untreated cultures at 4 h (Fig. 2a). Cultures were imaged by CLSM at 4, 7, and 22 h after bacterial inoculation. All phage-treated cultures were compared with untreated controls. To assess the viability of phage-treated cultures (see Fig. S5c), samples were removed, serially diluted, plated onto TSB agar plates, and incubated at 37°C for 24 h to calculate the number of CFU per milliliter.

### Exopolysaccharide mutant aggregate formation and dynamics in SCFM2.

Exopolysaccharide studies were performed with *P. aeruginosa* PAO1 Δ*pel* Δ*psl* and PAO1 Δ*psl* (25) containing pGJB5 (28) and PAO1 Δ*pel* and PAO1 Δ*algD* (25) containing pMRP9-1 (34). Experiments were performed identically to the established-infection protocol already described. Cultures were imaged by CLSM 22 h after bacterial inoculation. All mutant studies were compared with untreated controls that were prepared identically to samples treated with phage.

### Dispersal assay.

*P. aeruginosa* carrying pGJB5 was diluted to an OD_600_ of 0.05 in 250 µl of SCFM2 and added to microchamber wells. Aggregates were allowed to develop for 4 h at 37°C. After 4 h, 250 µl of fresh prewarmed (to 37°C) SCFM2 without bacterial cells was then gently pipetted on top of the SCFM2 containing preformed aggregates. Cultures were incubated statically at 37°C for a further 6 h and then imaged by CLSM. Imaris image analysis software was used to detect dispersed biomass in the initially uninoculated SCFM2 (this area corresponds to 60 to 100 µm above the base of the coverslip).

### Imaging aggregates.

All images were acquired with Zeiss LSM 700 and LSM 880 confocal laser scanning microscopes utilizing Zen image capture software. Bacterial cells were visualized via mCherry with an excitation wavelength of 587 nm and an emission wavelength of 610 nm or via GFP with an excitation wavelength of 488 nm and an emission wavelength of 509 nm with a 63× oil immersion objective. SCFM2 images were acquired by producing 512- by 512-pixel (0.26- by 0.26-µm pixels) 8-bit z-stack images that were 60 and 100 µm from the base of the coverslip for phage-treated/untreated experiments (Fig. 1 to 5) and the dispersal assay (Fig. 5b), respectively. The total volumes of the 60- and 100-µm z-stack images were 1,093.5 and 1,822.5 mm^3^, respectively. Control images of uninoculated SCFM2 were acquired by using identical settings to determine the background fluorescence for image analysis.

### Image analysis.

All imaging was performed with identical image capture settings. To determine the background fluorescence in SCFM2, a histogram of detected mCherry fluorescence was produced in Imaris v 8.3.1 (Bitplane) for uninoculated SCFM2, and the average of the three highest voxel values was determined as the background fluorescence. Averaging across all of the control images, this value was calculated as 81 voxels. This value was then subtracted from all experimental images with Imaris. The aggregate areas of *P. aeruginosa* from CF lung tissue that were used to calculate the aggregate cross-sectional area are from reference 18. For this study, a total of 61 peptide nucleic acid fluorescence *in situ* hybridization (FISH)-treated biofilms taken from 17 tissue specimens from three pairs of CF lungs were imaged. To calculate the 2D cross-sectional area of aggregates in SCFM2 from 3D confocal images, a random-number generator was used to choose three individual slices between 10 and 50 µm from the base of the coverslip at 7 and 22 h after *P. aeruginosa* inoculation. With Fiji image analysis software v 2.0.0, the background fluorescence was subtracted from the images. Borders were then drawn around visible aggregates with Fiji, and the analyze particles function was used to calculate individual cross-sectional areas. Aggregate sizes in SCFM2 were calculated from three biological replicate SCFM2 cultures with three slices from each to calculate the cross-sectional area of 40 aggregates per culture (120 aggregates in total).

### Calculation of aggregate dynamics in SCFM2.

For aggregate studies in SCFM2, isosurfaces were produced for all remaining voxels after background subtraction with the surpass module in Imaris. To detect individual aggregates, the split objects option in Imaris was enabled and aggregates were defined as objects with volumes of >5 µm^3^. The total biomass (all voxels detected), average aggregate volume, number of aggregates, and the z position were calculated within the vantage module in Imaris. Calculation of dispersed biomass in time-lapse experiments: To calculate the total dispersed biomass over time, isosurfaces were created as described above but from 3D CLSM stacks collected at 30-min intervals for 15 h. Detected aggregate isosurfaces were then ordered by volume. Objects that were ≥0.5 and ≤5.0 µm^3^ were categorized as dispersed biomass, and objects that were >5.0 µm^3^ were categorized as aggregated biomass. The total dispersed biomass was calculated as the sum of all of the dispersed objects in both untreated and treated cultures. For the dispersal assay, the ability of *P. aeruginosa* aggregates to colonize uninoculated SCFM2 was determined by calculating the total biomass of aggregates between 60 and 100 µm above the coverslip. Isosurfaces were created as described above to detect aggregates. All image data were exported into Microsoft Excel 2016, and graphs were generated with GraphPad Prism 7.

### Phage treatment of planktonic cultures.

*P. aeruginosa* carrying pGJB5 (28) was grown overnight in TSB at 37°C while shaking at 200 rpm. Cells were washed two times with PBS (pH 7.0). The OD_600_ was measured with a spectrophotometer, and washed bacterial cultures were inoculated into TSB at an OD_600_ of 0.05 (~10^7^ CFU/ml). Phage were added simultaneously with bacterial cells at a 2:1 phage-to-bacterium ratio. Cultures were vortexed for 5 to 10 s and incubated at 37°C with shaking at 200 rpm for 18 h. A 50-µl sample was then serially diluted and plated on TSB agar plates, and the number of CFU per milliliter was calculated. Phage-treated cultures were compared with untreated controls. Experiments were performed in triplicate.

### Statistical analysis.

Statistical tests performed were by analysis of variance (ANOVA), by two-tailed *t* test, randomization test, or by linear regression as stated in the figure legends. *t*, *F*, and *P* values for each test, as well as the degrees of freedom, are stated in each individual figure legend.
