# Supplementary figures and images for: Phage Inhibit Pathogen Dissemination by Targeting Bacterial Migrants in a Chronic Infection Model
Source: mBio. 2017 Apr 4;8(2):e00240-17. doi: 10.1128/mBio.00240-17 (PMC5380840; doi:10.1128/mBio.00240-17)

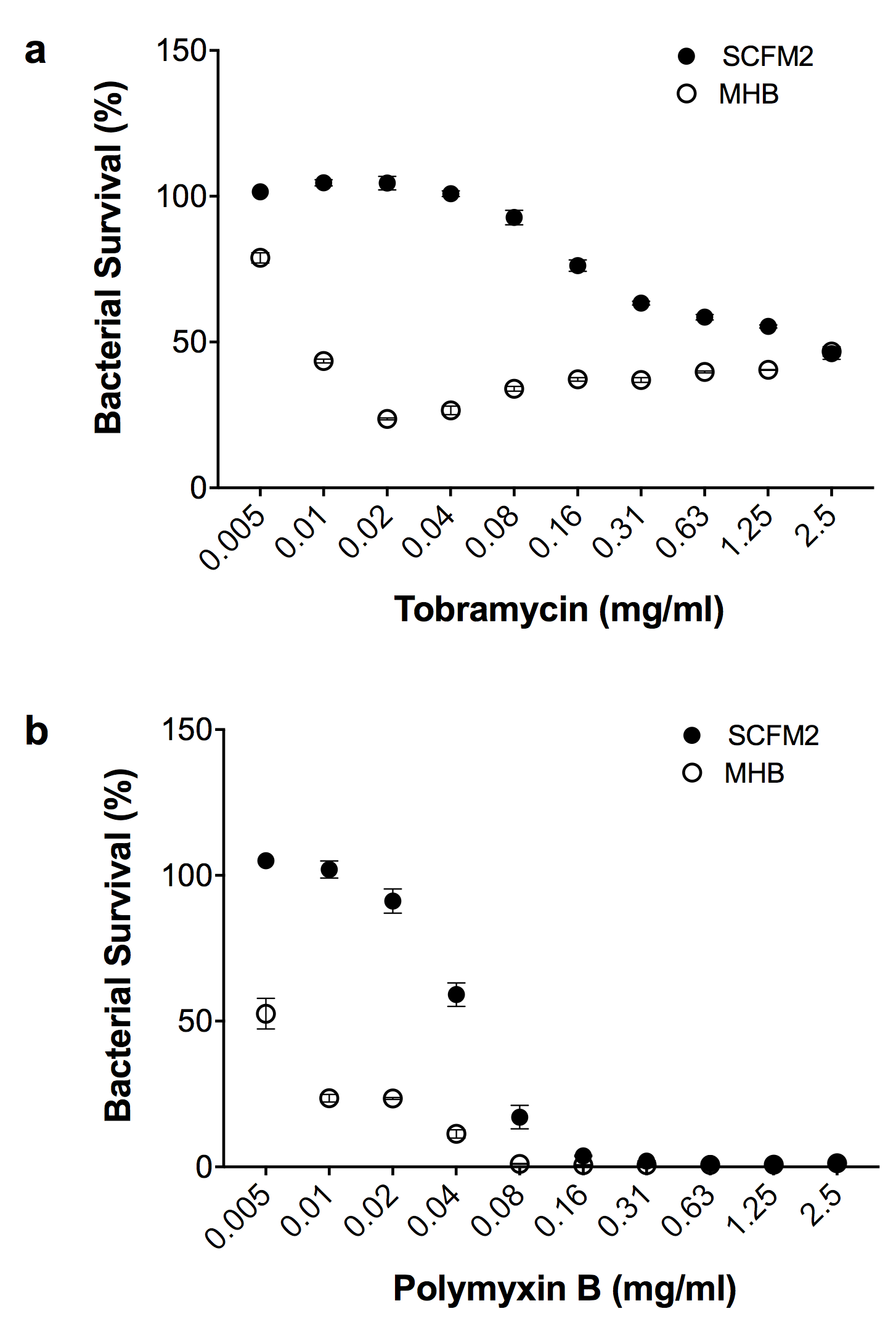

Supplement: FIG S1 [file mbo002173250sf1.tif]

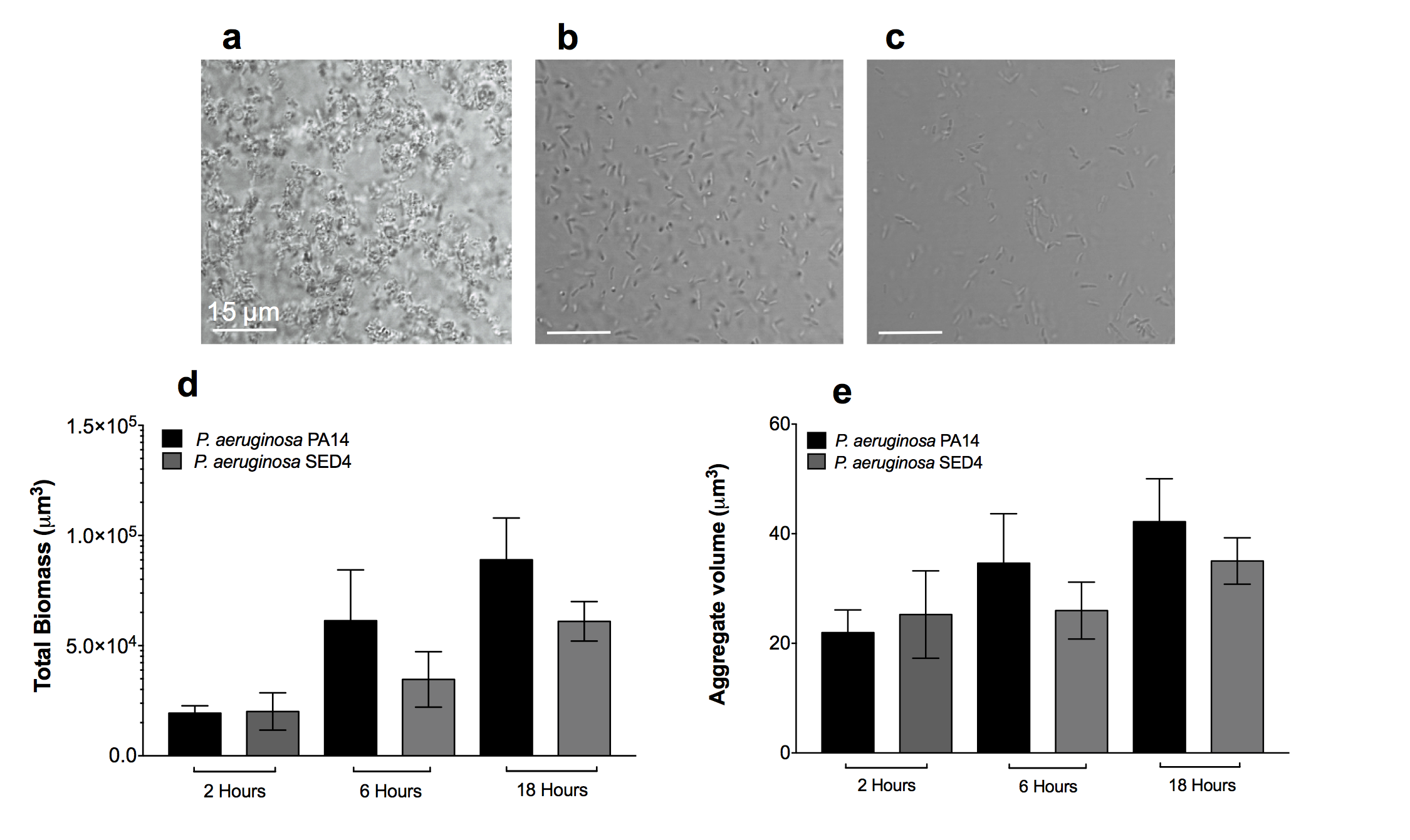

Supplement: FIG S2 [file mbo002173250sf2.tif]

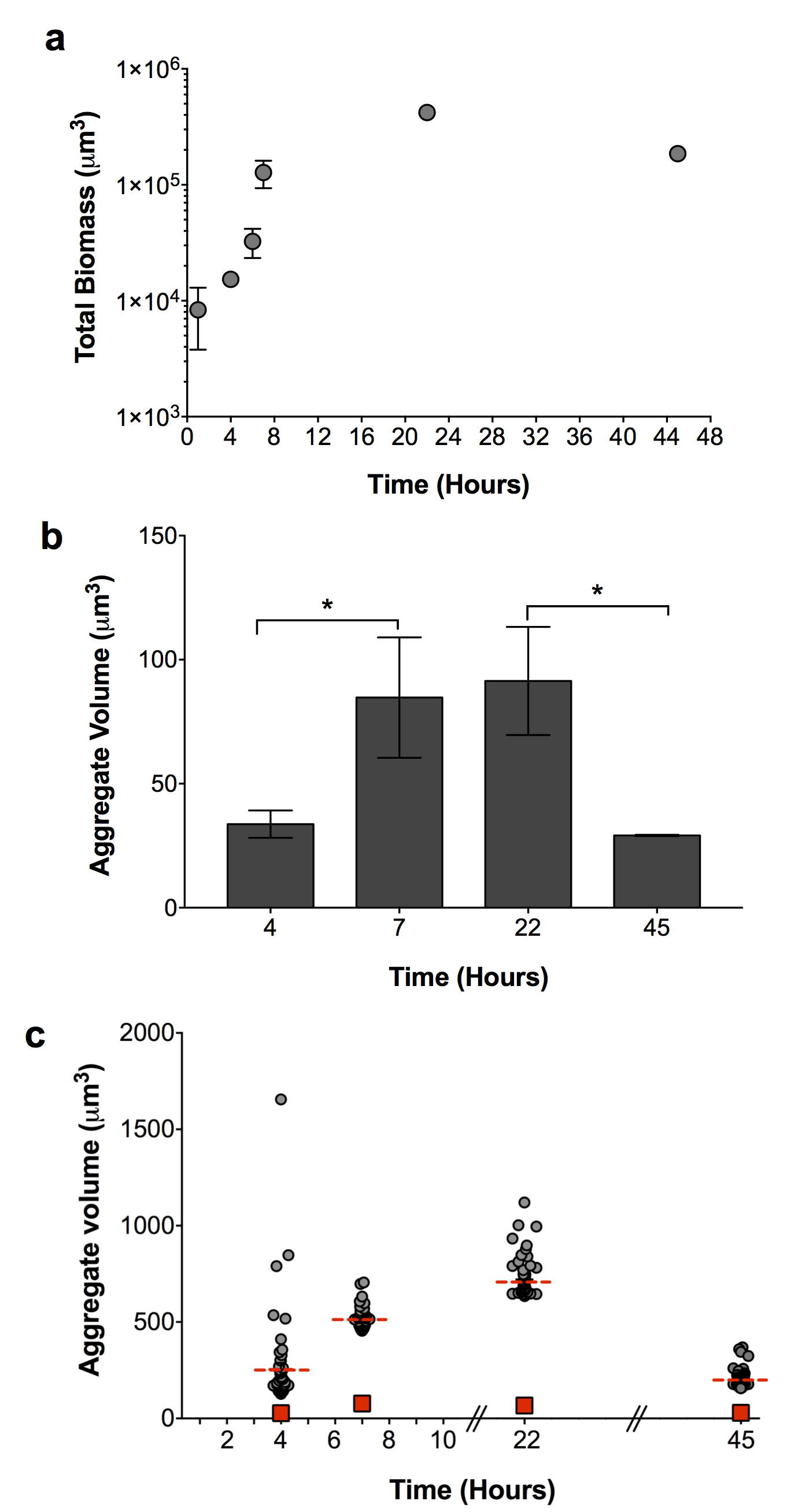

Supplement: FIG S3 [file mbo002173250sf3.tif]

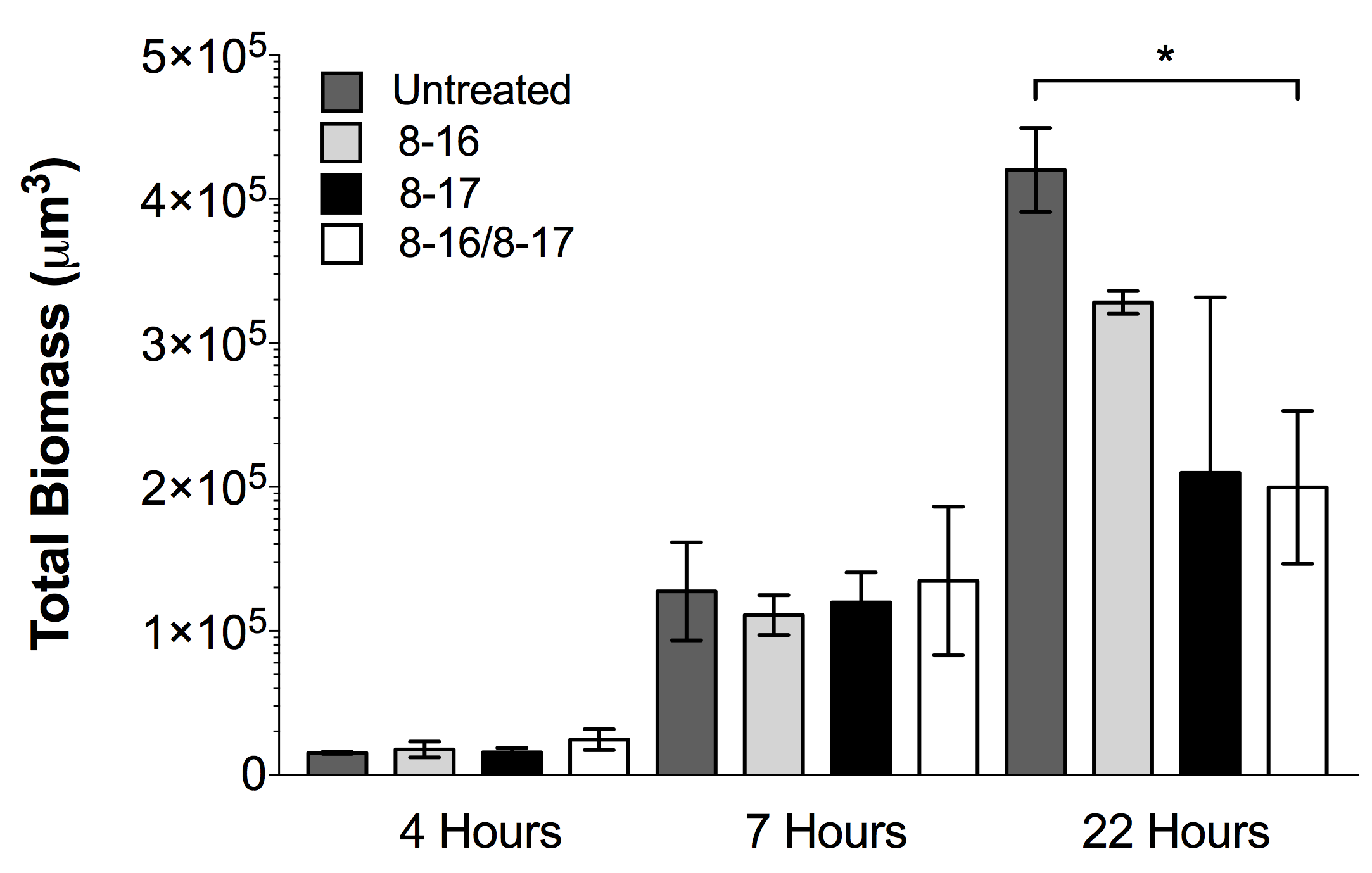

Supplement: FIG S4 [file mbo002173250sf4.tif]

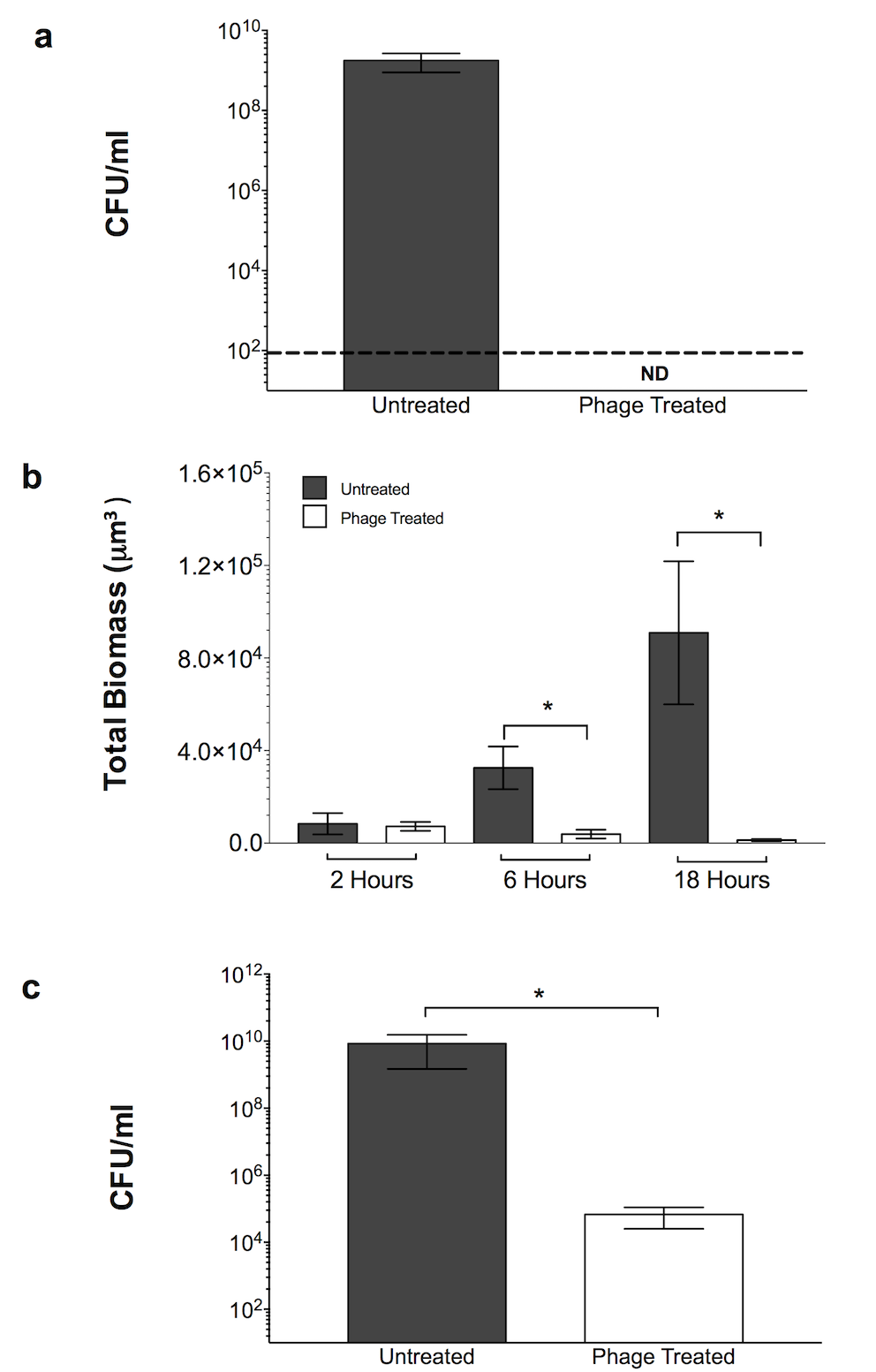

Supplement: FIG S5 [file mbo002173250sf5.tif]

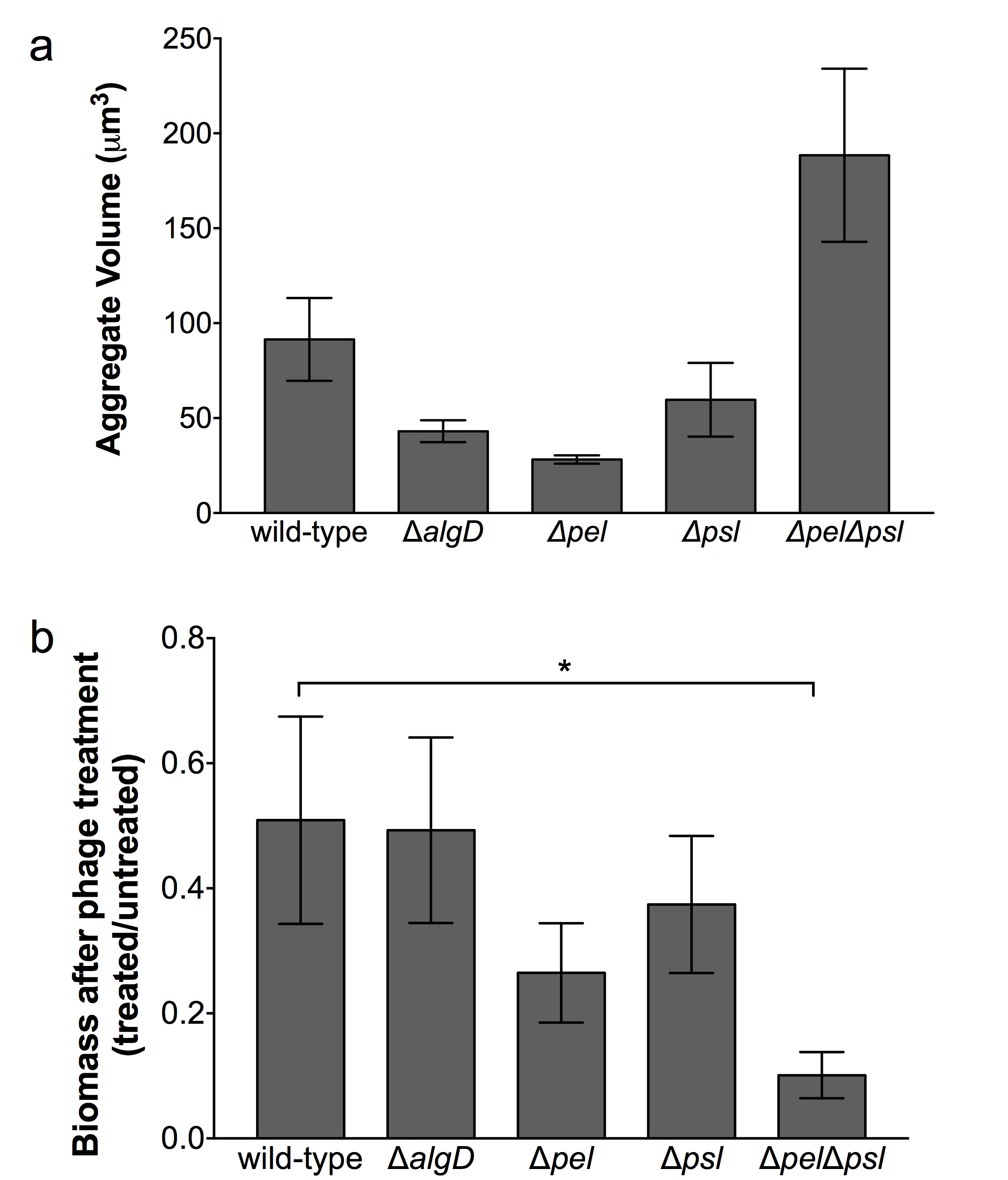

Supplement: FIG S6 [file mbo002173250sf6.tif]

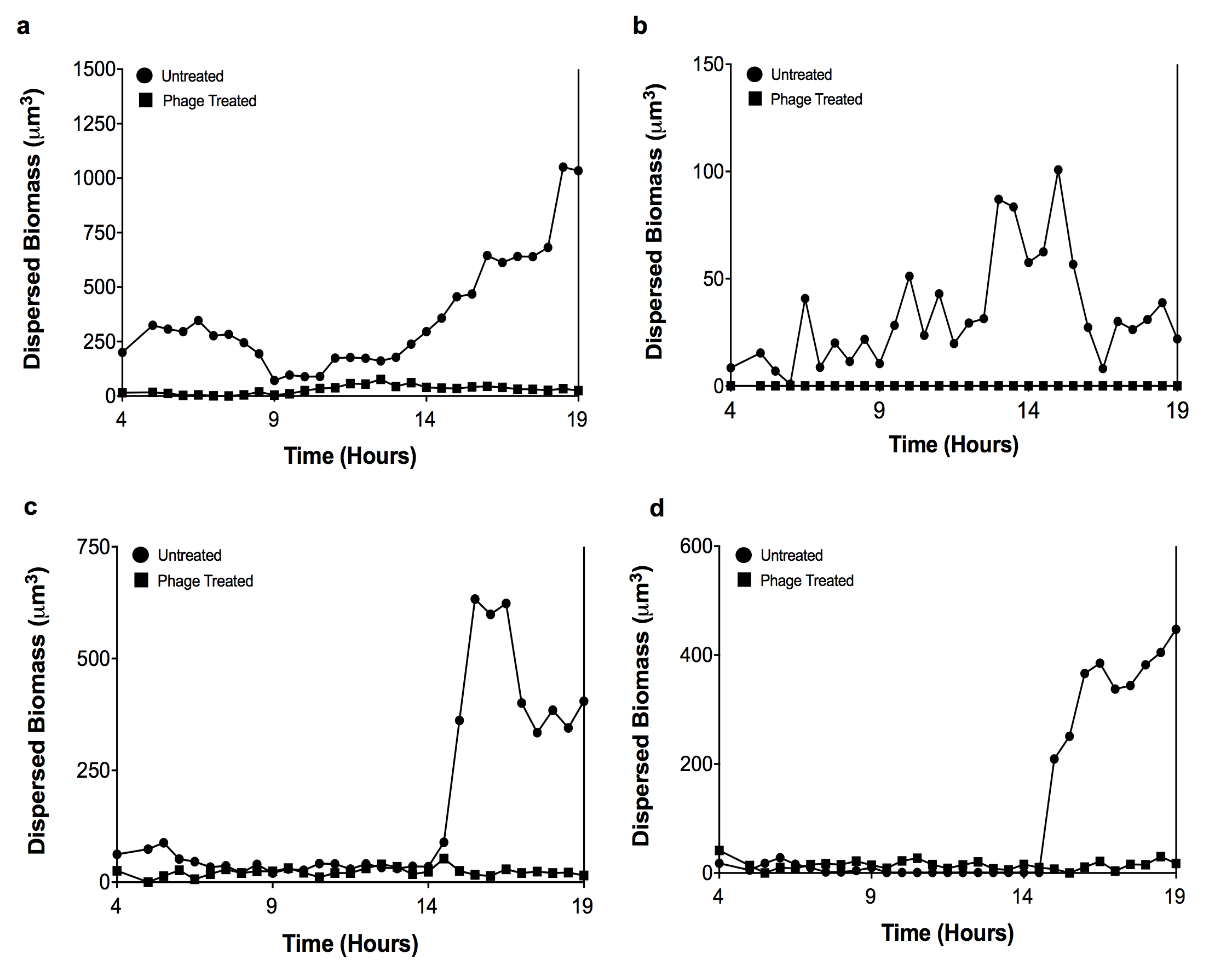

Supplement: FIG S7 [file mbo002173250sf7.tif]

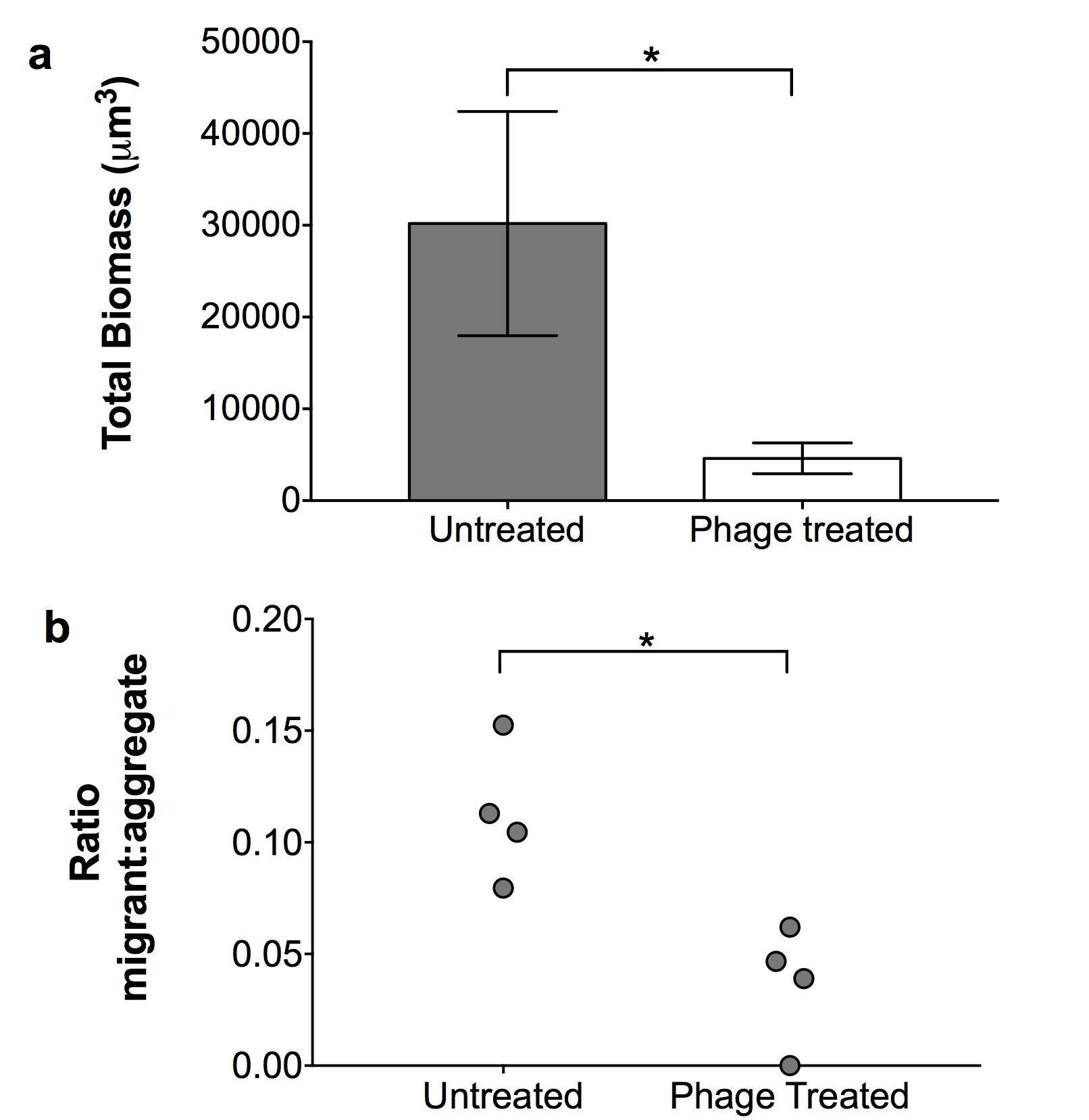

Supplement: FIG S8 [file mbo002173250sf8.tif]
